# Supplementary material for: TsRNA-49–73-Glu-CTC: A promising serum biomarker in non-small cell lung cancer
Source: PLoS One. 2025 Mar 28;20(3):e0320187. doi: 10.1371/journal.pone.0320187 (PMC11952254; doi:10.1371/journal.pone.0320187)
Supplement: S1 Table — (DOCX) [file pone.0320187.s001.docx]

| **Table S1 Patient characteristics** | | |
| --- | --- | --- |
| **Feature** | **Patient Count (n=32)** | **Percentage (%)** |
| **Gender** |  |  |
| -Man | 17 | 53.13% |
| -Female | 15 | 46.87% |
| **Age (Mean ± SD)** | 64.0±8.7 |  |
| **Pathology Type** |  |  |
| - Adenocarcinoma | 31 | 96.88% |
| - Adenosquamous Carcinoma | 1 | 3.12% |
| **TNM Stage** |  |  |
| - Stage I-II | 27 | 84.38% |
| - Stage III-IV | 5 | 15.62% |

SD：Standard Deviation; TNM Stage: Tumor-Node-Metastasis Stage
