# Supplementary figures and images for: TsRNA-49–73-Glu-CTC: A promising serum biomarker in non-small cell lung cancer
Source: PLoS One. 2025 Mar 28;20(3):e0320187. doi: 10.1371/journal.pone.0320187 (PMC11952254; doi:10.1371/journal.pone.0320187)

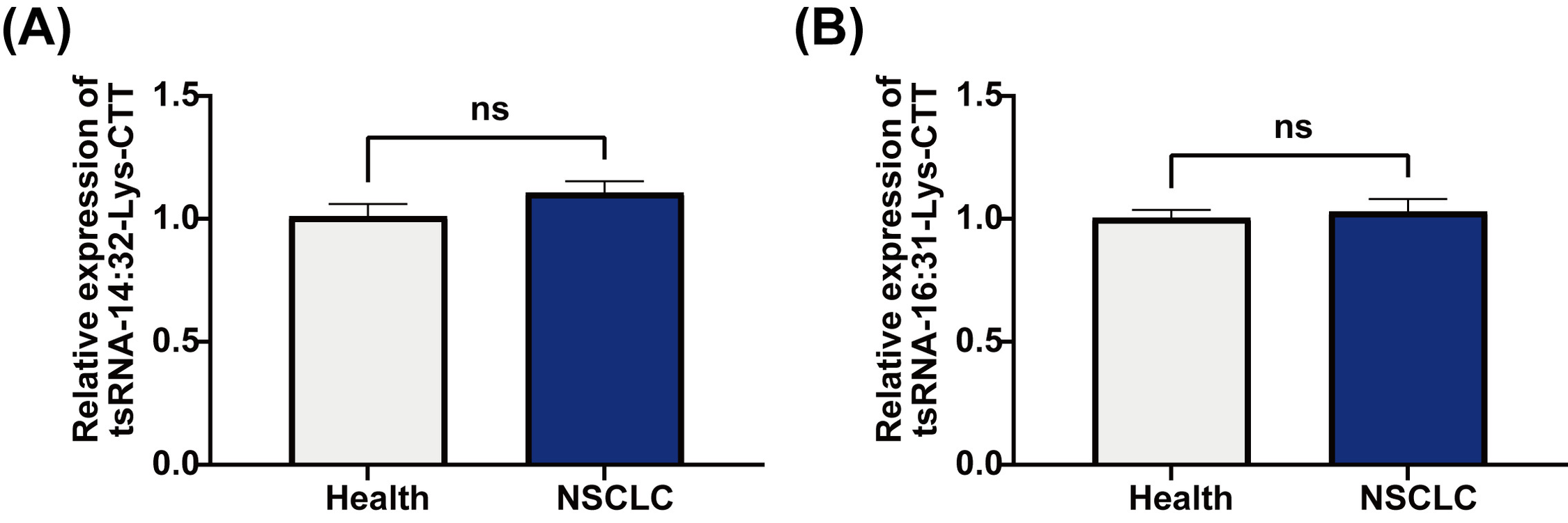

Supplement: S1 Fig — (TIF) [file pone.0320187.s002.tif]
